# Multiscale feature fusion for few-shot medical image learning with fisher information-driven layer selection

**DOI:** 10.1186/s42492-026-00220-6

**Published:** 2026-05-29

**Authors:** Kai Zhang, Yanjun Peng, Bo Pang, Xue Chen

**Affiliations:** 1 School of Computer Science and Engineering, Shandong University of Science and Technology, Shandong Province 266590 Qingdao, China; 2 Siemens Digital Industries Software, Wilsonville, OR 97070-7777 USA

**Keywords:** Few-shot learning, Low-rank adaptation, Medical image classification, Multiscale feature fusion, Parameter-efficient fine-tuning, Vision transformer, Fisher information

## Abstract

Few-shot medical image classification is a highly challenging problem in computer-aided diagnosis, with the central difficulty being enabling deep models to learn discriminative features conducive to classification from limited labeled samples. Vision transformers (ViTs) have recently demonstrated outstanding performance across various visual tasks. However, owing to their large parameter counts and dependence on massive pretraining data, ViTs are prone to overfitting in sample-scarce scenarios typical of few-shot learning. Parameter-efficient fine-tuning (PEFT) techniques, such as low-rank adaptation (LoRA), have alleviated some of these issues. However, conventional PEFT approaches still encounter difficulties in complex medical image classification tasks. To address this, this study proposes a general fine-tuning framework called a hierarchical probing and fusion network (HPF-Net), which integrates three core innovations to allow smarter and more efficient adaptation for few-shot medical image classification. First, a Fisher information-driven layer selection strategy strengthens the layer-selection robustness in few-shot settings. Subsequently, the attention-guided multiscale fusion module aligns and improves the features drawn from the selected critical layers. Subsequently, LoRA is incorporated into this efficient fine-tuning pipeline to reduce the parameter overhead while improving the accuracy. Extensive experiments on the public few-shot medical image benchmark, the medical imaging meta-dataset, demonstrated that HPF-Net significantly outperformed baseline methods, and ablation studies validated the necessity of each proposed component. The source code will be released upon acceptance.

## Introduction

Although public medical imaging benchmarks such as LiTS [1], HAM10000 [2], optical coherence tomograph datasets (Octs) [3], and DeepDRiD [4] have substantially advanced the field, high-quality labeled medical images remain considerably more difficult to obtain in clinical practice than natural images, particularly for rare diseases or imaging modalities that require expensive equipment, resulting in highly limited sample availability [5, 6]. This scarcity impedes the development and clinical deployment of automated computer-aided diagnosis systems, potentially increasing clinicians’ workload and reducing diagnostic efficiency. Therefore, developing deep learning models capable of effective learning from scarce data is of great clinical significance and practical value for improving diagnostic accuracy in rare conditions and promoting the use of artificial intelligence (AI)-assisted diagnosis. This study aimed to address this issue by constructing a highly adaptive and parameter-efficient model framework that can be rapidly deployed across a range of few-shot medical imaging tasks to provide clinicians with flexible and reliable decision support. The field of AI is undergoing a paradigm shift driven by foundation models [7]. Large language models and vision transformers (ViTs), including ViT [8], CLIP [9], DeiT [10], and MAE [11], exhibit remarkable generalizability and reasoning abilities after large-scale pretraining. However, this introduces practical challenges: training and adapting such massive models requires substantial computation, data, and time. For most research groups, full fine-tuning of models with tens or hundreds of billions of parameters is becoming increasingly impractical, particularly in vision, where ViTs, in contrast to convolutional neural networks built around residual connections [12] and batch normalization [13], lack built-in inductive biases such as translation equivariance and locality; consequently, ViTs rely heavily on large-scale visual corpora such as ImageNet [14] and COCO [15] to learn fundamental image properties and are prone to performance degradation and overfitting under limited data. Consequently, the development of more efficient model adaptation strategies has become a central problem driving progress in this field. Historically, this challenge has evolved into three phases. The initial phase is transfer learning: to avoid training from scratch for every new task, pretrained feature extractors (e.g., on ImageNet [14]) are typically frozen, and only the top classification layers are retrained, which is computationally inexpensive but lacks flexibility [16]. The next phase is few-shot learning (FSL): when labeled data are extremely limited, research shifts toward “learning to learn,” with methods such as model-agnostic meta-learning [17] and prototypical networks [18] that train across many related tasks to allow rapid adaptation to new tasks [19]. The subsequent phase is parameter-efficient fine-tuning (PEFT): as foundation models grow, the goal becomes adapting them without storing full model copies per task or incurring the high cost of full fine-tuning [20]. PEFT adjusts only a small fraction of the parameters (frequently $<$1%), with low-rank adaptation (LoRA) as a canonical example [21]. Complementary to PEFT is the design of lightweight backbones (e.g., MobileNets [22]) that reduce the number of parameters and FLOPs, providing practical advantages for deployment in resource-limited clinical environments.

PEFT methods aim to use pretrained models by updating only a small set of additional or localized parameters (typically under 1%), thereby achieving task adaptation with minimal computational and storage cost while maintaining performance close to full fine-tuning [20]. Current PEFT techniques can be categorized into four broad families based on how they modify pretrained model parameters. The additive family inserts small trainable modules into a frozen backbone, for example, adapter tuning [23]. Selective fine-tuning updates only specific parameter subsets in the pretrained model. Bias-term fine-tuning (BitFit), for instance, updates only the bias terms [24]. Prompt-based methods optimize additional learnable soft prompts appended to inputs, as exemplified by prompt tuning [25]. Reparameterization methods express weight updates in compact forms such as LoRA, which represents updates using low-rank decompositions [21]. Each family exhibits trade-offs in terms of parameter cost, inference overhead, and implementation complexity. LoRA has been widely used because of its zero inference-time latency overhead and efficient task-switching capability, spurring many follow-up variants.

LoRA’s core assumption is that task-specific weight changes $\Delta W$ during adaptation lie in a low-dimensional subspace [21]. LoRA parameterizes the weight updates as

$$W^{\prime} = W_0 + \Delta W = W_0 + BA$$

where $A \in \mathbb{R}^{r \times d_{in}}$ and $B \in \mathbb{R}^{d_{out} \times r}$ represent trainable low-rank matrices, and the original weight $W_0$ is frozen. This formulation requires updating only a small number of parameters during training and permits merging $BA$ into $W_0$ at the inference time, avoiding additional latency. This efficient mechanism makes LoRA practical for rapid task switching. LoRA also has the following limitations: the expressiveness of a fixed low-rank subspace can be insufficient, rank $r$ may not fit different tasks optimally, and quantized models may exhibit instability. Several extensions have been developed to address these issues. DoRA (weight-decomposed LoRA) decomposes weights into magnitude and direction and models only the direction with a low-rank update to improve flexibility [26]. LoRA with full-rank weight scaling augments low-rank updates with learnable scaling factors for more nuanced adjustments. Adaptive budget allocation for PEFT assigns adaptive ranks to layers based on their importance, whereas dynamic search-free low-rank adaptation allows the ranks to change during training. Quantized low-rank adaptation demonstrates the maintenance of high-quality fine-tuning when the backbone is quantized (e.g., 4-bit/8-bit) using specialized quantization formats and dual-quantization techniques, thereby reducing memory usage while preserving performance. Collectively, these variants retain the central PEFT philosophy–keeping the backbone frozen and updating a small number of parameters–while improving expressiveness, computational efficiency, and adaptation flexibility, advancing PEFT into a more systematic and hierarchical research direction.

Medical AI research commonly has limited sample sizes, posing long-standing challenges for model generalization [5, 6]. PEFT, particularly LoRA-style methods, provides a practical path for leveraging large pretrained models in few-shot medical image classification. Transformer-based architectures such as TransUNet, a Transformer-enhanced U-net for medical image segmentation, have also shown advantages in modeling structured features in medical imaging [27–29]. The medical imaging meta-dataset (MedIMeta) consolidates 10 medical domains, 19 imaging datasets, and 54 tasks and serves as a comprehensive benchmark for cross-domain FSL [30]. Deep models encode multiscale information across layers ranging from fine-grained to high-level semantics, a phenomenon previously observed in representation-visualization and attribution studies [31–33]. Linear probes were used to quantitatively assess the discriminative power of intermediate representations by training simple classifiers on a fixed-layer output [34]. Alain and Bengio [34] noted that inserting probes at multiple depths effectively determined whether a layer’s representation already contained useful information for distinguishing target classes. This observation is particularly relevant to medical imaging, where an accurate diagnosis frequently requires the combination of cues at multiple scales. However, selecting the most valuable layers and efficiently fusing them under tight parameter budgets remains an open problem. Supervised feature selection techniques, such as Fisher-score-based measures, provide a possible strategy [35]. Motivated by the above insights, the proposed hierarchical probing and fusion network (HPF-Net) unifies Fisher-driven dynamic layer selection, PEFT for ViTs, and intelligent multiscale fusion into a collaborative framework that enhances few-shot medical image classification while preserving the parameter efficiency.

Although PEFT techniques have achieved notable success in both vision and natural language domains, their application to few-shot medical image classification still faces challenges, particularly blind adaptation, in which adaptation strategies lack sensitivity to task-specific feature demands. To address this issue, the present study makes the following contributions.

- Fisher information-driven layer selection: a general fine-tuning mechanism with a dynamic layer selection strategy guided by Fisher information is proposed and validated. This provides precise guidance on applying PEFT to ViTs, addressing prior methods that adapt all layers blindly or rely on fixed subsets.
- Attention-guided multiscale fusion module: an intelligent fusion module is designed to align and combine features from different depths and improve generalization, producing superior results compared to simple combinations.
- LoRA-integrated efficient fine-tuning framework: global LoRA adaptation is combined with dynamic layer selection and multiscale fusion. The framework performs parameter-efficient adaptation across the backbone and selectively extracts and fuses features only from the layers most relevant to the current task, ensuring precise use of task-specific information.

## Methods

**Fig. 1:**
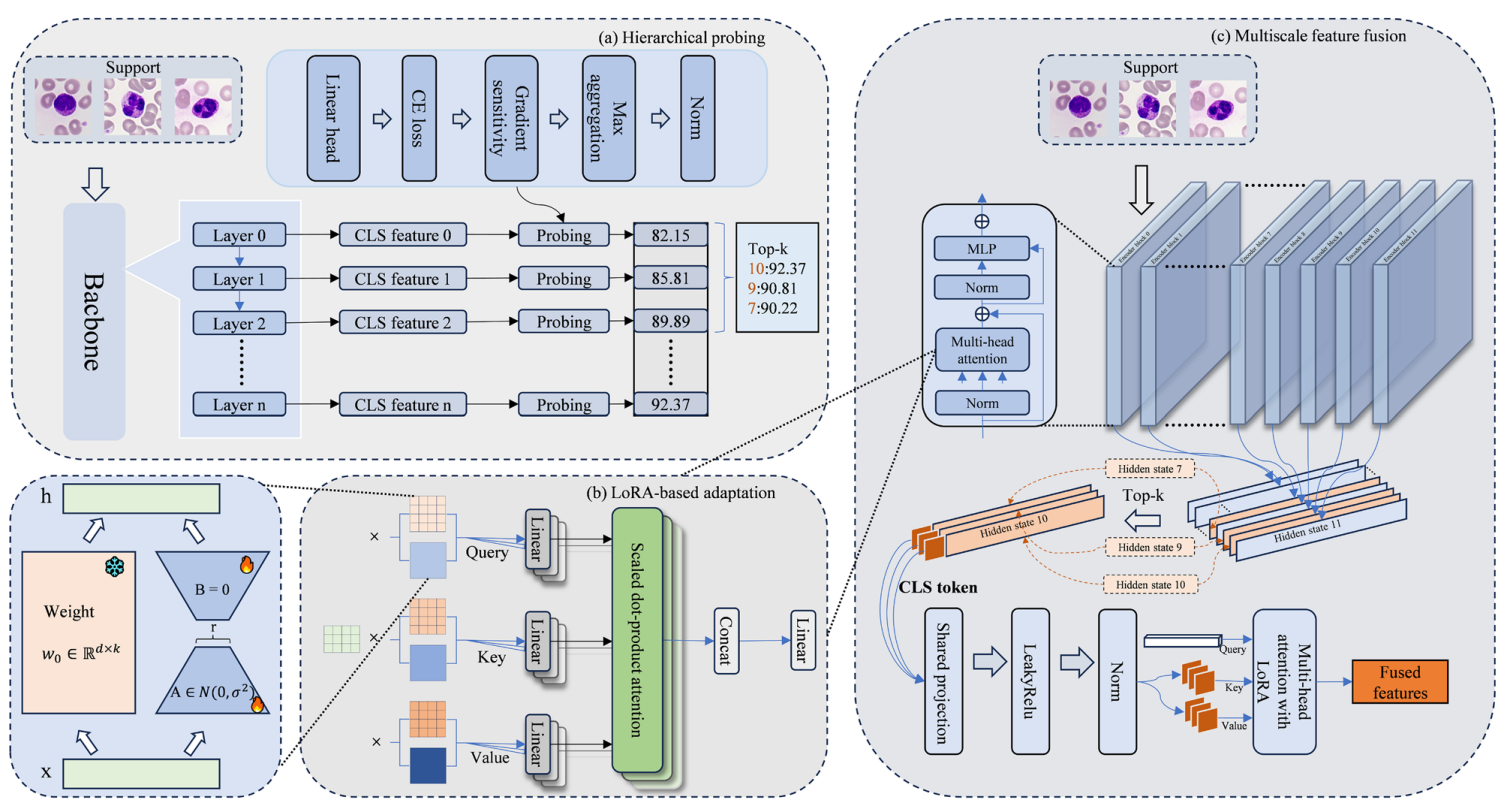
Overview of hierarchical probing and fusion network. **a** Fisher information-driven layer selection identifies task-relevant vision transformer layers; **b** Low-rank adaptation-based parameter-efficient adaptation updates the backbone through low-rank branches; **c** Attention-guided multiscale feature fusion aggregates selected layer features for final prediction

This section details the proposed HPF-Net, designed for few-shot medical image classification with a probe-then-fuse adaptation workflow. The core concept separates task-adaptive feature selection from parameter-efficient adaptation and unifies them through a fusion module. The framework consists of three main components: (1) Fisher information-based dynamic layer probing and selection, (2) a lightweight PEFT mechanism, and (3) an attention-guided multiscale feature-fusion module. Figure 1 shows an overview of the framework.

HPF-Net operates by splitting adaptation into probing/selection and fusion/adaptation phases. The probing phase evaluates the informativeness of the layer representations to identify those most relevant to the current task. The fusion phase constructs an efficient representation and fine-tunes it in a parameter-efficient manner. This separation clarifies which representations should contribute to the final decisions and how to optimize the information flow under parameter constraints, allowing the cooperative improvement of multilayer features.

### Notation and problem statement

Let the pretrained ViT backbone be $F(\cdot;\Theta)$ with $L$ transformer layers. The backbone follows the standard transformer design with layer normalization [36] and GELU activations [37]. For an input image $x$, the hidden state at layer $\ell$ is $H_\ell(x) \in \mathbb{R}^{T \times d}$, where $T$ denotes the number of tokens and $d$ denotes the feature dimensionality. This layer is represented as $f_\ell(x)$. If a [CLS] token is present, it is used as the representative feature $f_\ell(x) = H_\ell(x)[:,0] \in \mathbb{R}^d$; otherwise, average pooling is applied to the tokens. The few-shot task is an N-way K-shot problem with a support set $S = \{(x_i, y_i)\}_{i=1}^{NK}$ and query set $Q = \{(x_j, y_j)\}_{j=1}^M$. HPF-Net aims to efficiently use the support set $S$ such that the classification of the query set $Q$ is improved. The final fused discriminative feature is denoted as $F_{out}(x) \in \mathbb{R}^d$.

### Overall framework

HPF-Net follows a probe-select and fuse-adapt workflow that decomposes the adaptation into two linked stages. The probe-select stage quickly evaluates layer-wise informativeness and selects task-relevant layers. The fuse-adapt stage uses selected layers to build a parameter-efficient fusion structure and fine-tune it. This design separates the choice of feature sources from the parameter-constrained optimization of information flow, allowing for the cooperative improvement of multilevel representations.

### Fisher information-driven layer selection

Medical imaging tasks rely on feature representation at different depths. Some tasks require shallow-layer details, such as textures and edges, whereas others require higher-level semantics. The use of a fixed set of layers across tasks is suboptimal. To address this issue, a Fisher information-based dynamic layer selection mechanism was introduced to identify the most discriminative layer representations for each task.

This mechanism uses the classical Fisher score concept for all layer outputs. For each layer $\ell$, the goal is to measure how well $f_\ell(x)$ separates classes in the support set $S$. The support samples $(x_i, y_i)$ are processed through the frozen ViT backbone to cache all the layer outputs $\{f_\ell(x_i)\}_{\ell=1}^L$ without any training. A randomly initialized linear classification head was temporarily attached to each layer, cross-entropy loss on the support set was computed, and gradients of the loss with respect to the layer features were obtained. The squared gradients were averaged per class to derive a quantity analogous to the Fisher score, reflecting the sensitivity of the layer representation to class distinctions. For each layer, the maximum of these class-wise Fisher values was considered as the layer score $\mathrm{score}_\ell$. The layers were ranked by $\mathrm{score}_\ell$ and the top $K_s$ layers formed the selected fusion set $\mathcal{S}_{selected}$. This procedure demonstrates robustness under few-shot and class-imbalance settings because it directly quantifies the feature contributions to classification.

To formalize, consider the classical Fisher score for a feature dimension $j$ as follows:

1 $$S_j = \frac{\sum_{c=1}^{C} n_c (\mu_{cj} - \mu_j)^2}{\sum_{c=1}^{C} n_c \sigma_{cj}^2}$$

where $\mu_{cj}$ and $\sigma_{cj}^2$ represent the mean and variance of feature $j$ for class $c$, respectively, $\mu_j$ represents the global mean, and $n_c$ denotes the number of samples in class $c$ [35]. Although effective for individual features, directly applying this formulation to high-dimensional layer representations $f_\ell(x) \in \mathbb{R}^d$ is impractical in few-shot settings because estimating per-dimensional statistics from $K$ samples (e.g., $K=1$ or $K=5$) is highly unreliable.

Instead, this study uses a gradient-based Fisher proxy that operates on entire layer representations. For layer $\ell$, a temporary linear classification head $h_\ell$ with a fixed random initialization was attached to the layer output. For each class $c$ with support subset $\mathcal{S}_c = \{(x_i, y_i) \in \mathcal{S} : y_i = c\}$, the classwise Fisher proxy is computed as follows:

2 $$\mathcal{F}_c^{(\ell)} = \frac{1}{|\mathcal{S}_c|} \sum_{x_i \in \mathcal{S}_c} \left\| \nabla_{f_\ell(x_i)} \mathcal{L}_{\mathrm{CE}}\bigl(h_\ell(f_\ell(x_i)),\, y_i\bigr) \right\|_2^2$$

where $\mathcal{L}_{\mathrm{CE}}$ denotes cross-entropy loss. This quantity corresponds to the diagonal empirical Fisher information (the expected squared gradient of the log-likelihood with respect to the layer representation) and measures the sensitivity of the classification loss to the layer output for class $c$.

The per-layer score aggregates class-wise proxies using the maximum as follows:

3 $$\mathrm{score}_\ell = \max_{c \in \{1,\ldots,C\}} \mathcal{F}_c^{(\ell)}$$

Considering the maximum ensures that a layer is selected if it is highly informative for distinguishing at least one class, which is preferable to mean aggregation under a class imbalance. The scores are then z-score normalized across layers for comparability.

4 $$\hat{s}_\ell = \frac{\mathrm{score}_\ell - \bar{s}}{\sigma_s + \epsilon}$$

where $\bar{s}$ and $\sigma_s$ denote the mean and standard deviation, respectively, of $\{\mathrm{score}_\ell\}_{\ell=1}^L$, and $\epsilon$ denotes a small constant for numerical stability.

#### Robustness under few-shot conditions

Several design choices render the scoring procedure reliable under few-shot conditions. Each layer’s temporary classification head uses a fixed random initialization shared across all classes so that class-wise Fisher proxies are directly comparable within a layer; z-score normalization then yields comparable scores across layers with different activation scales. Because the gradient-based proxy relies on first-order gradients rather than full covariance estimation, it remains well defined even with a single sample per class.

**Figure:**
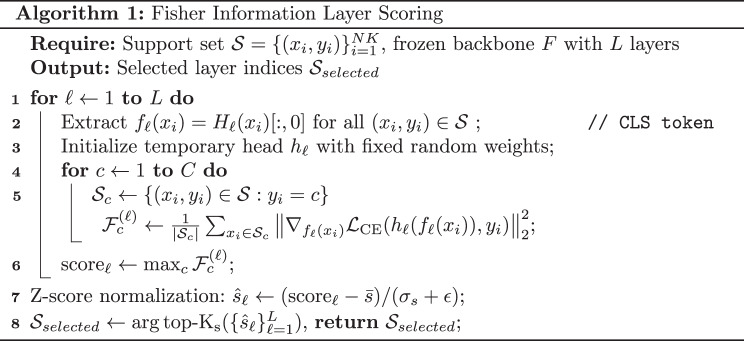

### PEFT and training strategy

With the selected layer set $\mathcal{S}_{selected}$, the full ViT backbone is adapted, rather than only updating a few layers. LoRA modules are injected into the query, key, and value projection matrices of each transformer layer. For any original weight matrix $W_0 \in \mathbb{R}^{d_{out} \times d_{in}}$, LoRA adapts as

$$W^{\prime} = W_0 + \Delta W$$

with

$$\Delta W = BA, \quad A \in \mathbb{R}^{r \times d_{in}},\; B \in \mathbb{R}^{d_{out} \times r}$$

and rank $r \ll \min(d_{in}, d_{out})$. This global adaptation provides extra tunable capacity throughout the feature extraction path to better match the downstream statistics with limited data.

During fusion and adaptation, the training objective minimizes the total classification loss $L_{total}$ of support set $S$. Let $\Theta_{\mathrm{lora}}$ denote all LoRA parameters, $\{A,B\}$, $\Theta_{\mathrm{fusion}}$ be the fusion module parameters, and $\Theta_{\mathrm{head}}$ be the final classifier parameters. The trainable parameter set is

$$\Theta_{\mathrm{trainable}} = \Theta_{\mathrm{lora}} \cup \Theta_{\mathrm{fusion}} \cup \Theta_{\mathrm{head}}$$

The optimization problem can be written as

$$\min_{\Theta_{\mathrm{trainable}}} L_{total} = L_{\mathrm{CE}}\left(H\left(G(S;\mathcal{S}_{selected},\Theta_{\mathrm{lora}});\Theta_{\mathrm{fusion}}\right);\Theta_{\mathrm{head}}\right)$$

where $G(\cdot)$ denotes the backbone and feature extraction process after LoRA injection, $H(\cdot)$ denotes the fusion and classification, and $L_{\mathrm{CE}}$ denotes the cross-entropy loss.

All original ViT weights outside $\Theta_{\mathrm{trainable}}$ remain frozen, ensuring that the downstream adaptation relies on LoRA branches, multiscale fusion module, and classifier. Differential learning rates are applied: the classification head learning rate is scaled with respect to task complexity (number of classes $N$) to accelerate boundary formation on more complex tasks, whereas LoRA parameters use a smaller, steadier learning rate for stability. The optimization uses AdamW with a cosine annealing learning rate schedule and gradient clipping to improve stability under few-shot conditions.

### Attention-guided multiscale feature fusion

**Fig. 2:**
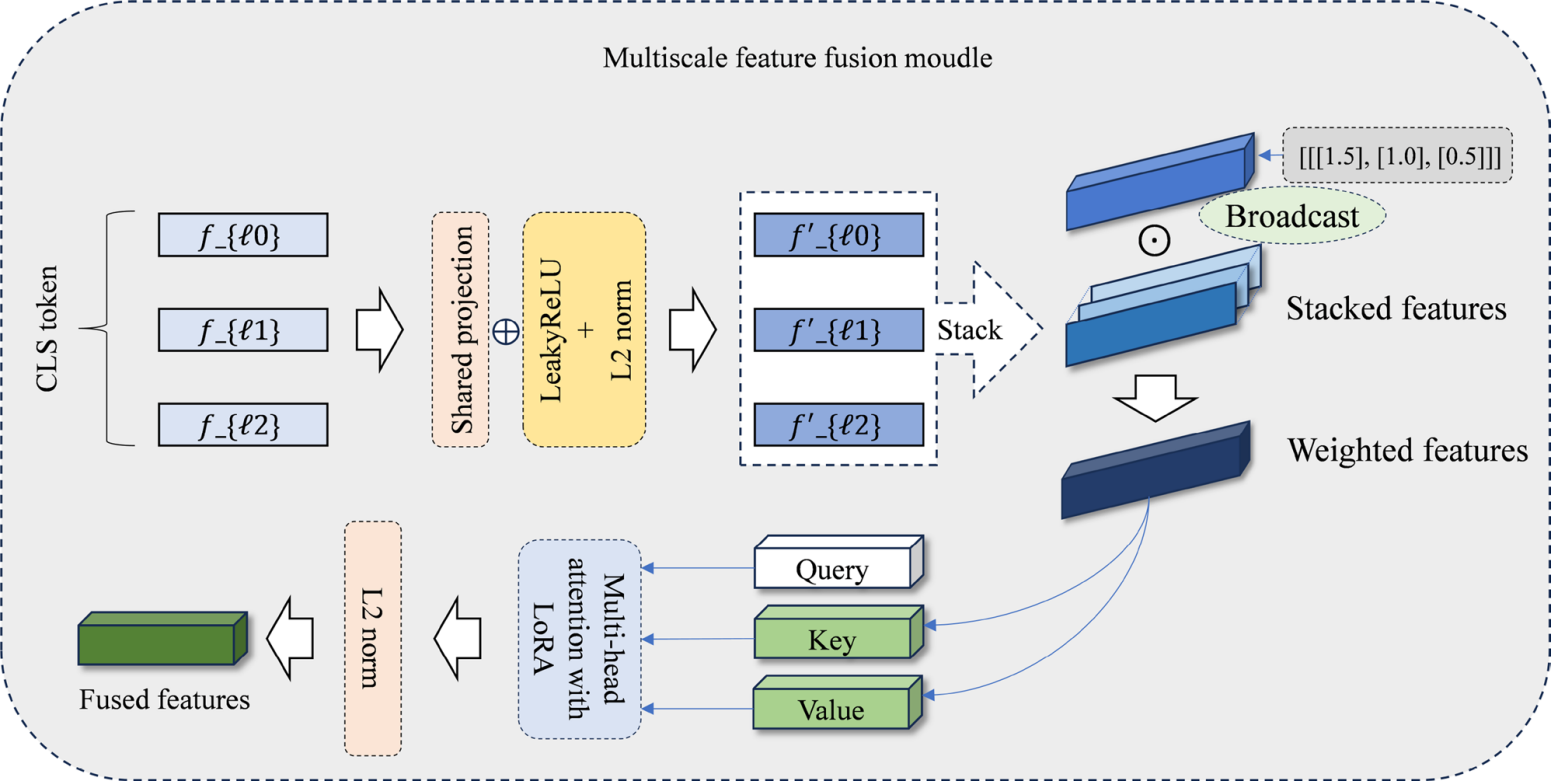
Detailed architecture of the attention-guided multiscale feature fusion module. Features from the selected top-K layers are first aligned in a unified space using a shared projection layer. The fusion is then performed using a dual-weighting mechanism that combines learnable static layer weights with dynamic multi-head attention to aggregate features according to sample-specific characteristics

The fusion module integrates features from LoRA-adapted backbone-selected layers. It consists of a shared projection layer for feature alignment, static layer-weighting mechanism, and dynamic attention-based aggregation unit. This dual-weighting scheme combines a stable prior with a sample-specific judgment to achieve robust and flexible feature fusion, as shown in Fig.  2.

Given an input image, the features $\{f_\ell(x)\}_{\ell\in\mathcal{S}_{selected}}$ are extracted and projected onto a shared space, followed by a Leaky Rectified Linear Unit (LeakyReLU) activation and L2 normalization.

$$\tilde{f}_\ell(x) = \frac{\mathrm{LeakyReLU}(P_{\mathrm{shared}} f_\ell(x) + b_p)}{\|\mathrm{LeakyReLU}(P_{\mathrm{shared}} f_\ell(x) + b_p)\|_2}$$

The shared projection maps features from different depths to a unified representation, reducing parameter redundancy and serving as a structural regularization that mitigates overfitting in scarce data regimes.

Static layer weighting uses learnable scalars $\{w_\ell\}_{\ell\in\mathcal{S}_{selected}}$ initialized with a linearly decreasing sequence and updated during training, yielding

$$\hat{f}_\ell(x) = w_\ell \cdot \tilde{f}_\ell(x)$$

Stacked-scaled feature form

$$F_{\mathrm{stack}} = [\hat{f}_{l_1}(x),\ldots,\hat{f}_{l_{K_s}}(x)]^\top \in \mathbb{R}^{K_s\times d}$$

and are queried by a learned input-agnostic vector $q\in\mathbb{R}^{1\times d}$. Multi-head attention computes dynamic weights and aggregates information using

$$\mathrm{Attention}(q,F_{\mathrm{stack}},F_{\mathrm{stack}})=\mathrm{softmax}\left(\frac{qK^\top}{\sqrt{d_k}}\right)V$$

where $K=F_{\mathrm{stack}}W_K$ and $V=F_{\mathrm{stack}}W_V$. The attention output $F_{\mathrm{fused}}^{\prime}$ is then passed through a LeakyReLU activation and L2 normalization to produce the final fused feature

$$F_{\mathrm{out}}(x)=\frac{\mathrm{LeakyReLU}(F_{\mathrm{fused}}^{\prime})}{\|\mathrm{LeakyReLU}(F_{\mathrm{fused}}^{\prime})\|_2}$$

L2 normalization projects features onto the unit hypersphere; therefore, the classifier operates in the feature direction rather than the magnitude.

#### Implementation details

Table  1 lists the architectural hyperparameters of the fusion module. The shared projection is a single linear layer $P_{\mathrm{shared}} \in \mathbb{R}^{d \times d}$ applied identically to all selected layers, followed by LeakyReLU activation (negative slope of 0.1) and L2 normalization. The static layer weights $\{w_\ell\}$ are initialized with a linearly decreasing sequence from 1.5 to 0.5, reflecting a prior favoring deeper layers. The fusion query $q \in \mathbb{R}^{1 \times d}$, initialized using the Xavier normal distribution, attends to the stacked-layer features through a cross-attention mechanism with eight heads and dropout [38].

**Table 1:** Architectural hyperparameters of the attention-guided fusion module

Component	Setting
Shared projection	Linear($d$,$d$) + LeakyReLU(0.1) + L2 normalization
Static layer weight	Learnable, init: linspace(1.5, 0.5,$K_s$)
Fusion query	Learnable$q \in \mathbb{R}^{1 \times d}$, Xavier normal init
Multi-head attention	8 heads, dropout = 0.1, cross-attention
Post-attention	LeakyReLU(0.1) + L2 normalization

### Algorithm

Algorithm 2 (HPF-Net) summarizes the entire pipeline for N-way K-shot tasks. Given task samples and a pretrained backbone, the algorithm outputs predictions on the query set. Fisher information was used to probe and score all backbone layers for their discriminative contributions and to select the top-ranked layers (detailed in Algorithm 1). Low-rank LoRA adapters were injected into the backbone for parameter-efficient adaptation and attention-guided multiscale fusion of aggregate-selected-layer features. Training updated only lightweight components (LoRA, fusion module, and classifier), significantly reducing the parameter overhead while preserving expressivity.

**Figure:**
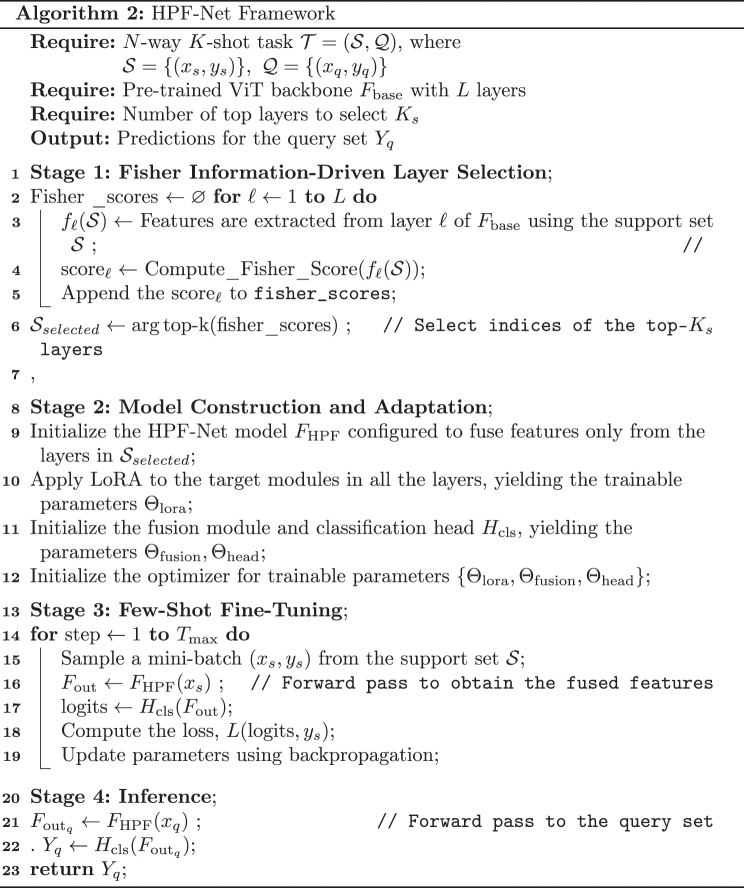

### Experimental setup

To comprehensively evaluate HPF-Net, experiments were conducted using the MedIMeta multidomain metadataset [30]. Four representative datasets were selected: acute myeloid leukemia cell morphology (Aml) [39], dermoscopy (Derm) [2], the Oct  [3], and diabetic retinopathy grading (Dr_regular) [4]. All experiments followed the standard N-way K-shot meta-learning protocol, with N equal to the number of classes in each dataset and K set to 1, 5, and 10 to simulate the varying degrees of sample scarcity. The area under the receiver operating characteristic curve (AUROC) was used as the primary evaluation metric because of its robustness to class imbalance and its ability to reflect overall discrimination across decision thresholds.

The baselines included a diverse set of strong competitors: full-tuning (updating all model parameters) as an upper-bound reference; linear probing (freezing the backbone and training only a linear classifier) as a parameter-efficient baseline; partial-1 (selective fine-tuning of the final transformer block) to evaluate localized adaptation; MLP-3 (a three-layer MLP classifier on frozen features) to assess a stronger classification head; and mainstream PEFT methods such as Bias (BitFit) [24], AdaptFormer [40],  shallow/deep visual prompt tuning (VPT-S/VPT-D) [41], embedded prompt tuning (EPT) [42], and standard LoRA [21].

All models used the google/vit-base-patch16-224-in21k pretrained backbone [8] and were optimized using AdamW [43, 44]. For HPF-Net, the default selected-layer count was $K_s=3$, a value determined by preliminary experiments that showed stable and strong overall performance across tasks and datasets. LoRA rank was fixed at $r=8$. The reported results were averaged over 100 randomly generated tasks. Hyperparameters for all methods were tuned independently using a grid search on the validation splits to ensure a fair comparison. Experiments were performed using NVIDIA RTX 4090 GPUs.

## Results and Discussion

### Main results

To systematically compare HPF-Net with the baselines, evaluations were performed across all datasets and three few-shot settings. Table  2 summarizes the results. HPF-Net achieved the best performance in nearly all settings, with an average AUROC of 80.93%, outperforming full fine-tuning by 17.30% points (pp), standard LoRA by 2.87 pp, and the next-best baseline (AdaptFormer, 78.20%) by 2.73 pp.

**Table 2:** Comparison of hierarchical probing and fusion network with various baseline methods on four medical imaging datasets, based on area under the receiver operating characteristic curve (%)

Method	Aml			Derm			Oct			Dr_regular			Average
Shot	1	5	10	1	5	10	1	5	10	1	5	10	
Full [16]	60.99	73.55	78.43	59.11	69.26	72.42	51.82	56.36	58.21	57.25	61.81	64.33	63.63 (-13.90%)
Linear [45]	73.38	87.31	91.02	63.07	75.60	81.38	60.03	73.70	80.12	60.10	68.38	72.70	73.90 (0.00%)
Partial-1 [16]	78.29	89.19	92.42	63.58	76.82	82.76	58.38	73.04	78.59	59.01	68.83	72.56	74.46 (0.76%)
MLP-3	78.64	89.44	92.80	64.04	76.99	82.71	58.20	72.80	79.02	59.07	69.11	72.56	74.62 (0.97%)
VPT-S [41]	78.84	88.92	92.13	63.91	77.41	83.38	60.79	74.44	80.47	59.94	69.27	73.26	75.23 (1.80%)
VPT-D [41]	77.28	91.19	95.17	63.82	78.35	84.88	61.91	77.37	83.74	62.91	70.61	74.66	76.82 (3.95%)
EPT [42]	77.63	91.55	95.11	64.02	77.77	83.59	64.56	79.62	85.38	64.33	72.72	**77.25**	77.79 (5.26%)
LoRA [21]	78.62	91.62	95.33	65.03	80.46	86.03	63.54	80.73	85.85	61.03	71.94	76.53	78.06 (5.63%)
Bias [24]	77.47	91.42	95.00	64.61	79.40	83.96	61.31	76.58	84.18	61.15	70.37	74.28	76.64 (3.71%)
AdaptFormer [40]	78.56	91.54	95.15	65.82	80.74	86.11	64.03	79.88	85.13	62.76	72.46	76.26	78.20 (5.82%)
HPF-Net (this study)	**81.45**	**94.80**	**97.07**	**68.84**	**83.52**	**88.16**	**66.96**	**83.30**	**88.97**	**66.96**	**73.88**	77.21	**80.93 (9.51%)**

Values in parentheses in the Averagecolumn denote relative changescompared with Linear probing, andboldface indicates the best result ineach column

### Ablation studies

The ablation studies quantified the contribution of each proposed component and reported the average AUROC across the four datasets. Table  3 reports the effectiveness of the dynamic layer selection against three fixed selection strategies: front k, evenly spaced k, and back k layers. Fisher-driven adaptive selection consistently outperformed any fixed strategy, indicating that different medical tasks preferentially used representations at different depths and that the dynamic mechanism could capture such preferences.

**Table 3:** Ablation study on the layer selection mechanism (area under the receiver operating characteristic curve (%), averaged over 1, 5, and 10-shot)

Configuration	Aml	Derm	Oct	Dr_regular	Avgerage
HPF-Net (Fisher)	**91.11**	**80.17**	**79.74**	**72.68**	**80.93**
First k	86.18	77.84	74.27	71.21	77.38
Spaced k	89.61	79.70	77.68	71.65	79.66
Last k	89.94	79.80	79.52	72.00	80.32

The boldface indicates the best result in each column

Table  4 summarizes the results of the fusion-module ablation study. Replacing attention-based fusion with simpler alternatives (average pooling or concatenation followed by a linear layer) led to considerable performance drops, demonstrating the superiority of attention-guided fusion for aggregating multiscale features.

**Table 4:** Ablation study on the fusion module (area under the receiver operating characteristic curve (%), averaged over 1, 5, and 10-shot)

Configuration	Aml	Derm	Oct	Dr_regular	Avgerage
Attention	**91.11**	**80.17**	**79.74**	**72.68**	**80.93**
AvgPool	90.28	78.43	78.37	71.90	79.75
Concatenation	90.37	78.06	78.11	71.66	79.55

The boldface indicates the best result in each column

Table  5 compares normalization choices in the fusion module under the 5-shot setting. L2 and L1 normalization performed comparably (83.88% *vs* 83.46%), whereas removing the normalization entirely caused a substantial decrease to 78.85%. These results indicate that projecting features onto a common scale before attention is important for stable fusion, although the specific norm type has a limited impact.

**Table 5:** Ablation study on normalization type in the fusion module (5-shot, area under the receiver operating characteristic curve (%))

Norm type	Aml	Derm	Oct	Dr_regular	Avgerage
L2 (this study)	**94.80**	**83.52**	**83.30**	**73.88**	**83.88**
L1	93.72	83.07	83.23	73.80	83.46
None	89.94	77.50	77.70	70.25	78.85

The boldface indicates the best result in each column

### Efficiency analysis

Because all methods share the same ViT-base backbone with nearly identical per-pass FLOPs, wall-clock runtime is reported rather than FLOPs to capture the actual efficiency differences. The learning rate $\in \{$1e-5, 1e-4, 5e-4, 1e-3, 2e-3, 5e-3$\}$ and training steps $\in \{$30, 50, 100, 150$\}$ were grid-searched independently for each baseline, and the runtime was reported under each method’s optimal configuration.

Table  6 lists the results. HPF-Net trained 1.11 M parameters (1.29% of the backbone), comparable to LoRA (0.45 M) and AdaptFormer (0.59 M), and the trainable parameter counts were independent of the shot setting. Compared to AdaptFormer (AUROC 81.16%), HPF-Net improved by 2.72% points at a comparable runtime (11.25 s *vs* 9.19 s under the 5-shot setting).

**Table 6:** Comparison of parameter efficiency, runtime, and performance across methods (5-shot, area under the receiver operating characteristic curve (%))

Method	Trainable parameter (M)	Trainable parameter (%)	Time/task (s, 5-shot)	AUROC (%)
Full-Tuning	85.80	100.00	9.27	65.25
Linear Probing	0.005	0.01	0.20	76.25
Partial-1	7.09	8.27	5.67	76.97
MLP-3	0.46	0.54	6.15	77.09
VPT-S	0.01	0.02	7.69	77.51
VPT-D	0.10	0.11	9.42	79.38
Bias (BitFit)	0.13	0.15	11.62	79.44
EPT	0.24	0.28	7.75	80.42
LoRA	0.45	0.52	11.33	81.19
AdaptFormer	0.59	0.69	9.19	81.16
HPF-Net (this study)	1.11	1.29	11.25	**83.88**

Runtime is wall-clock time per task on an NVIDIA RTX 4090, averaged over 100 tasks. The runtime of HPF-Net includes Fisher probing overhead. The boldface indicates the best result in each column

**Fig. 3:**
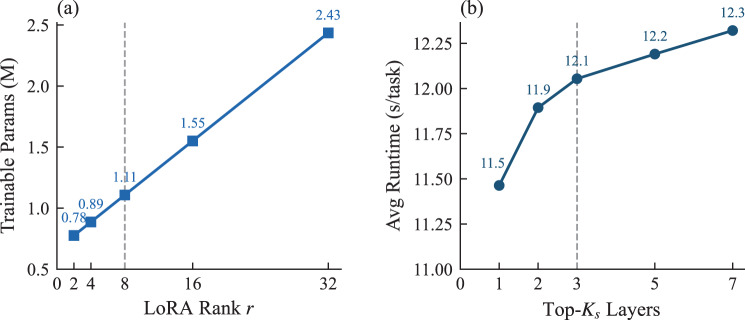
Computational cost of the architectural hyperparameters in hierarchical probing and fusion network. **a** Trainable parameters as a function of low-rank adaptation rank $r$ (with $K_s{=}3$); **b** Average per-task runtime as a function of the number of selected layers $K_s$ (with $r{=}8$). Gray dashed lines indicate default values

Table  7 further breaks down the per-task runtime of HPF-Net into three stages: Fisher probing, training, and evaluation. The probe time remained nearly constant across shot settings (0.30–0.37 s) because it performed a single forward-backward pass per layer regardless of the support size, whereas the training time increased with the number of samples. Consequently, the probe ratio decreased from 6.73% (1-shot) to 1.78% (10-shot), confirming that Fisher-based layer selection is a lightweight, fixed-cost operation that does not become a bottleneck as the support set scales up. Figure 3 shows how the computational cost scales with the two architectural hyperparameters: LoRA rank $r$ linearly increased trainable parameters, whereas the number of selected layers $K_s$ increased runtime owing to a larger fusion input.

**Table 7:** Stage-wise runtime breakdown of hierarchical probing and fusion network across shot settings (seconds per task, NVIDIA RTX 4090, averaged over 100 tasks)

Shot	Probe (s/task)	Train (s/task)	Eval (s/task)	Total (s/task)	Probe ratio (%)
1	0.30	4.07	0.09	4.46	6.73
5	0.32	10.83	0.09	11.25	2.88
10	0.37	20.22	0.09	20.68	1.78

### Hyperparameter sensitivity

This subsection examines sensitivity to the two architectural hyperparameters of HPF-Net: the number of selected layers $K_s$ and LoRA rank $r$. Both were fixed across all datasets and shot settings ($K_s{=}3$, $r{=}8$) without per-dataset tuning. The sensitivity sweeps were evaluated over 50 tasks with a fixed random seed to ensure reproducible pairwise comparisons; the main results in Table  2 are averaged over 100 randomly sampled tasks, which account for the minor numerical differences between the two settings.

**Fig. 4:**
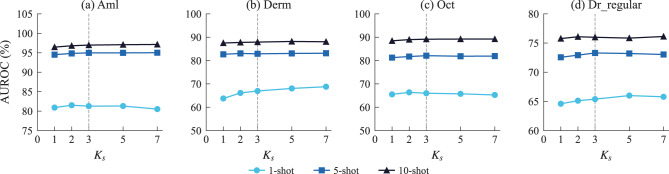
Sensitivity analysis for the number of selected layers $K_s$ across (**a**) Aml, (**b**) Derm, (**c**) Oct, and (**d**) Dr_regular under 1-, 5-, and 10-shot settings. Gray dashed line indicates the default value $K_s{=}3$

For $K_s$, a sweep over $\{1,2,3,5,7\}$ was conducted with $r{=}8$ fixed (Fig. 4). Using a single layer ($K_s{=}1$) consistently degraded the performance (79.50% *vs* 80.25% at $K_s{=}3$), which confirmed that multiscale fusion was beneficial. Performance plateaus for $K_s \geq 3$ with less than 0.1 pp variation; learnable weighting likely attenuated less-informative layers. $K_s{=}3$ was used as the default.

**Fig. 5:**
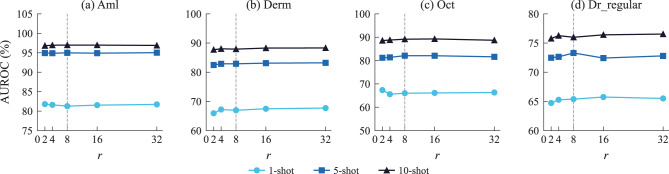
Sensitivity analysis for low-rank adaptation rank $r$ across (**a**) Aml, (**b**) Derm, (**c**) Oct, and (**d**) Dr_regular under 1-, 5-, and 10-shot settings. Gray dashed line indicates the default value $r{=}8$

For $r$, a sweep over $\{2,4,8,16,32\}$ was conducted with $K_s{=}3$ fixed (Fig. 5). AUROC remained stable across all datasets: the cross-dataset average ranged from 79.98% ($r{=}2$) to 80.37% ($r{=}32$), a variation of only 0.39% points, while the trainable parameters increased from 0.78 M to 2.44 M. The default $r{=}8$ (1.11 M) balanced accuracy and parameter cost well.

### Qualitative analysis

**Fig. 6:**
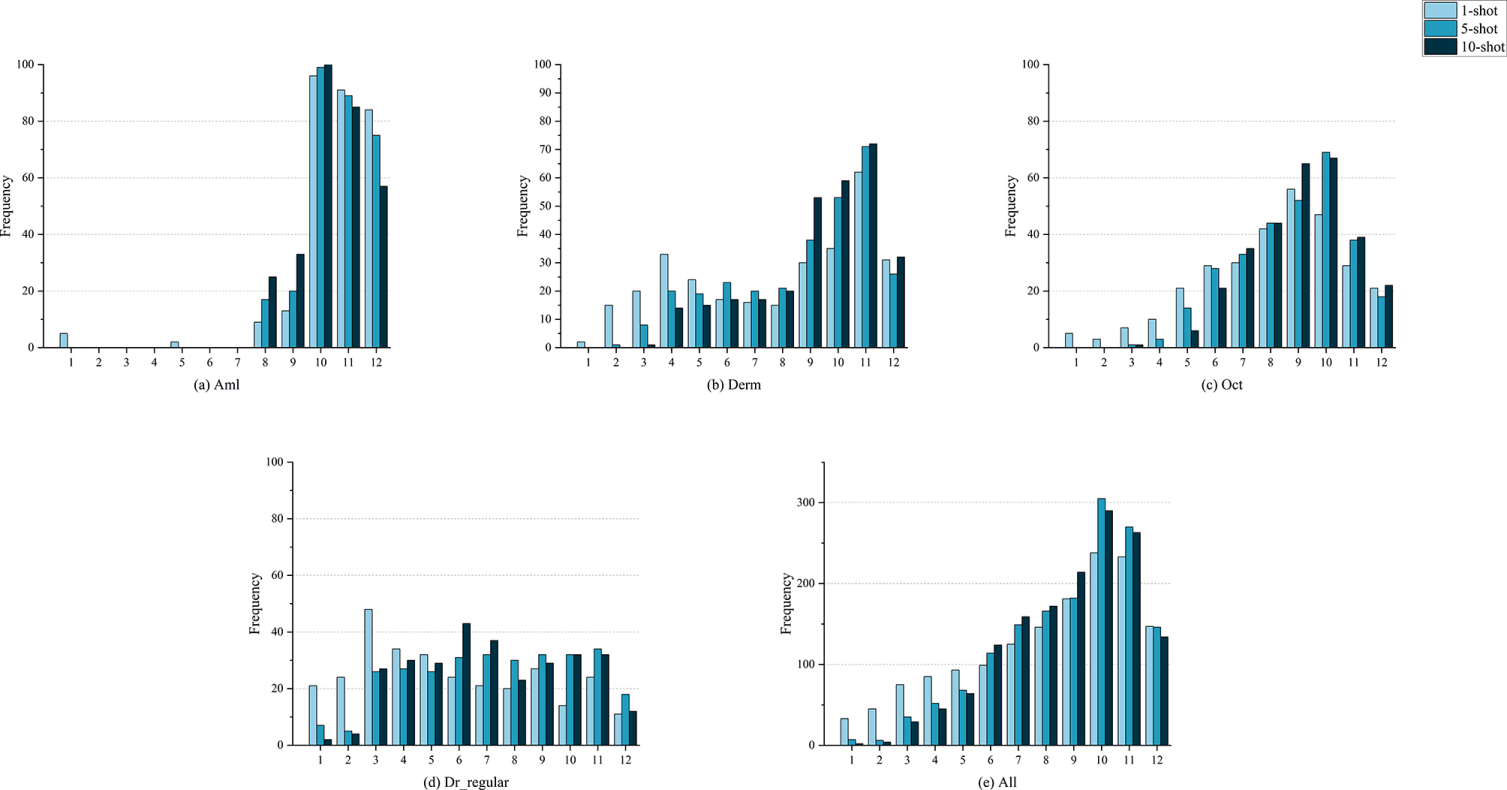
Layer selection frequencies based on Fisher information. This figure contains five subplots corresponding to (**a**) Aml, (**b**) Derm, (**c**) Oct, (**d**) Dr_regular, and (**e**) all tasks. Each subplot shows the total number of times each of the 12 ViT layers (1-12) was selected under 1-, 5-, and 10-shot settings

Visualization of layer selection frequencies (Fig.  6) reveal the behavioral patterns of the dynamic selector. Counting how frequently each ViT layer is selected from among the top K across all test tasks shows dataset- and shot-dependent preferences, with an overall tendency favoring mid-to-high layers (approximately layers 6–12), indicating that these depths frequently contain richer discriminative information. The variability of the preferred layers further motivates dynamic selection rather than a fixed selection policy.

**Fig. 7:**
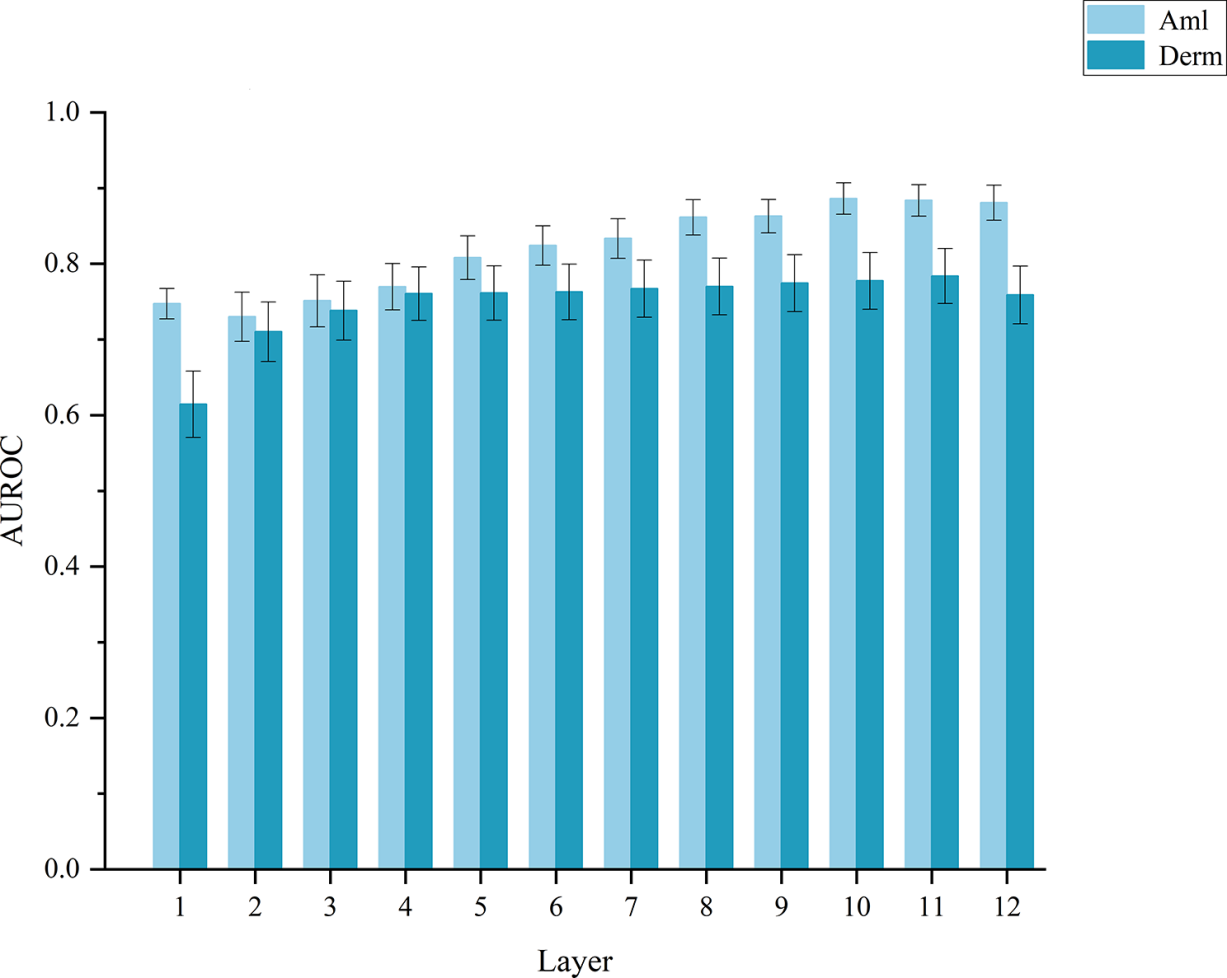
Linear probe evaluation of layer-wise representations. By training a linear classifier on the features of each layer, their discriminative power for different tasks can be assessed. The plot shows that classification performance varies significantly across layers and datasets, highlighting the requirement for task-adaptive layer selection

The linear probe evaluation of the per-layer representations (Fig.  7), by training a simple classifier on each layer and plotting the performance across depths, shows that the best-performing layer varies significantly across tasks: texture-focused tasks (e.g., Derm) frequently peak at middle depths, whereas tasks requiring more abstract understanding (e.g., cell morphology) tend to peak at deeper layers. Such task-dependent depth variations support the necessity of the proposed Fisher-driven dynamic selection.

**Fig. 8:**
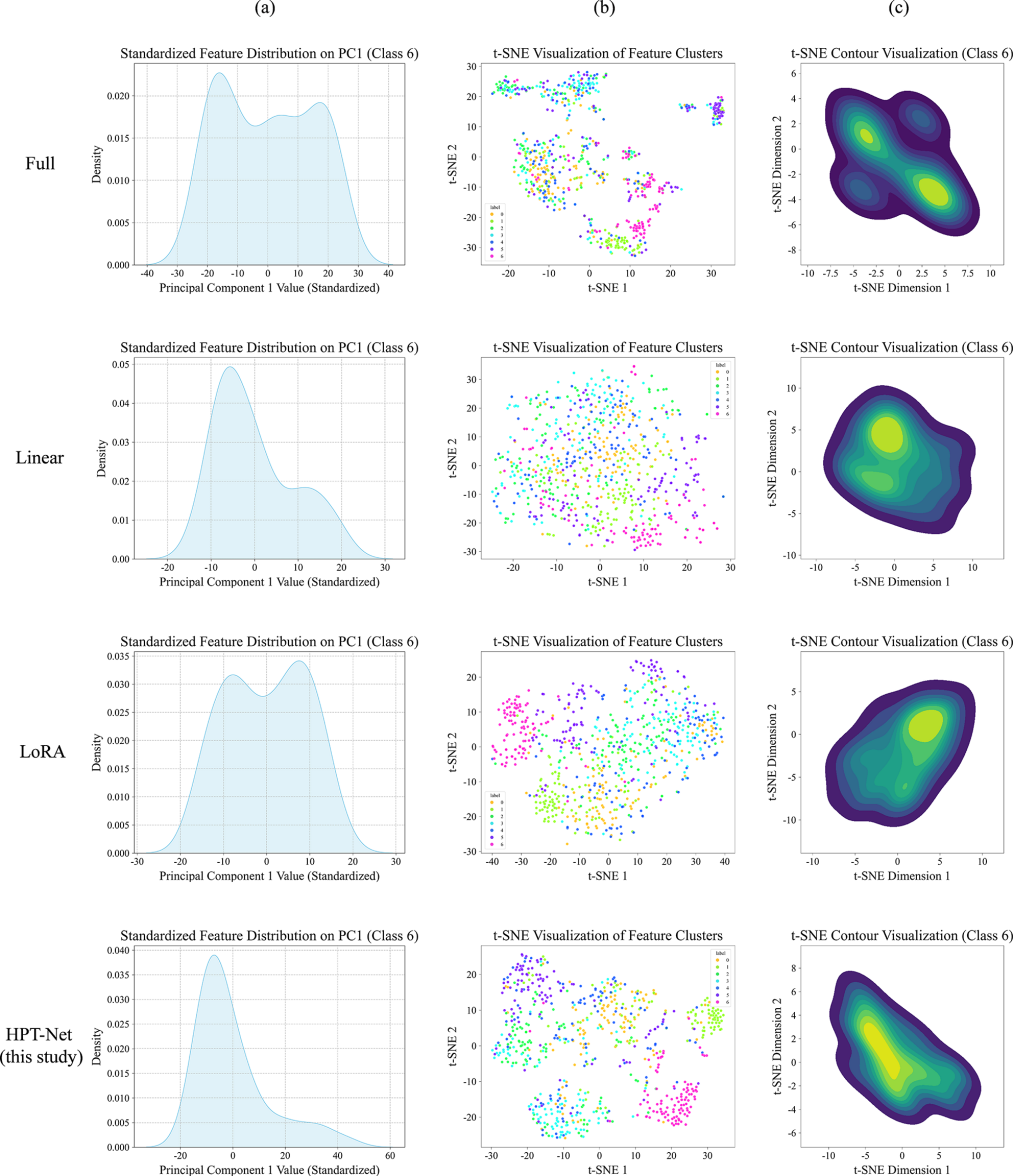
Comparative visualization of learned feature representations across methods under the 5-shot setting. **a** Principal-component distributions of same-class samples, showing intra-class compactness; **b** *t*-SNE 2D projection of multi-class features, showing inter-class separability; **c** *t*-SNE density contour plots for intra-class features, providing an alternative view of class compactness. Each panel compares full-tuning, linear probing, low-rank adaptation, and hierarchical probing and fusion network under identical experimental conditions; tighter clusters and clearer inter-class boundaries indicate superior feature quality

Figure 8 compares the learned representations across the methods along three axes: within-class compactness, between-class separability, and class-wise density, using PCA projections and *t*-SNE density contours. HPF-Net representations exhibited tighter within-class clusters, clearer between-class boundaries, and more unimodal, concentrated density regions than the baselines, suggesting purer and more compact class representations in the learned feature space.

### Robustness of hierarchical semantic properties

The hierarchical semantic properties of deep networks underpin HPF-Net. Linear probe analyses indicate that shallow layers (approximately layers 1–5) encode low-level texture patterns, edges, and local intensity changes, whereas deeper layers (approximately layers 9–12) capture more abstract concepts, such as organ contours or lesion types. In few-shot medical imaging, this hierarchy shows cross-domain robustness: although retinal scans from Dr_regular and dermoscopic images from Derm differ in high-level semantics, they share low-level texture components (for example, micro-boundaries and noise patterns), allowing shallow features to transfer across domains. Fisher-driven selection harnesses this robustness by detecting the abstraction level that is most discriminative for the current task, switching adaptively between texture-oriented shallow layers and semantics-oriented deep layers to avoid negative transfer from unrelated pretrained high-level semantics.

### Comparison with integrated spatial attention-style innovations

Contrasting HPF-Net with attention-based advances, such as integrated spatial attention or typical spatial/channel attention mechanisms used in medical imaging, highlights the key differences. Integrated spatial attention-type methods generally refine features within a fixed layer set, highlighting where (spatial) or what (channel) the focus is in the existing feature maps. Although effective for segmentation or large-scale classification, such methods remain static in the depth dimension and assume that the selected layers already contain the required information. HPF-Net introduces a dynamic depth-selection dimension, rather than refining all or a predetermined subset of layers indiscriminately. The probe-then-adapt paradigm questions whose depths are most informative for the specific few-shot task and can discard irrelevant or noisy layers before fusion. This selective allocation of computational resources is particularly important under conditions of data scarcity and reduces overfitting risk, creating a distinct optimization path compared with to conventional spatial/channel attention.

### Failure case analysis

Although HPF-Net achieved the best performance in nearly all settings, it marginally underperformed EPT on Dr_regular under 10-shot (77.21% *vs* 77.25%). Dr_regular is a diabetic retinopathy grading task with an ordinal class structure, where class boundaries represent subtle gradations of disease severity. The deep prompt tokens in EPT may capture such fine-grained sequential patterns more effectively, thereby reducing the relative benefit of multiscale fusion.

The Fisher-driven layer selection in HPF-Net can also exhibit instability in 1-shot settings, where the Fisher proxy is estimated from a single sample per class. With only one gradient observation, the estimated layer scores have a high variance, and the selected layers may not be generalized to the query set. This is reflected in the larger performance gaps between the 1-shot and 5-shot settings compared to the baselines. Fixed-head initialization and max-over-class aggregation mitigate, but do not eliminate, this limitation.

When inter-class visual differences are subtle (e.g., morphological differences in cell classification), Fisher score variation across layers diminishes, narrowing the advantage of dynamic selection over fixed strategies such as last k (80.93% *vs* 80.32% in Table  3). Thus, the benefit of dynamic selection is the most pronounced for tasks with heterogeneous feature demands across depths.

## Conclusions

### Summary

HPF-Net was proposed to alleviate the adaptation bottleneck of large pretrained models for few-shot medical image classification. Following a probe-then-adapt strategy, the framework used Fisher information for dynamic layer selection to identify discriminative layers per task, applied global LoRA for parameter-controlled adjustment of the backbone, and used an attention-driven fusion module to intelligently aggregate multiscale representations. Extensive benchmarks indicated that HPF-Net consistently outperformed the representative methods. Ablation studies and representation visualizations further demonstrate the independent contributions and complementarity of the proposed components, underlining the framework’s advantages in terms of adaptive efficiency and feature quality.

### Limitations and future work

Despite significant empirical improvements, several limitations remain that suggest directions for future research. The Fisher-driven probing step, while effective, introduces additional computational overhead, which motivates the exploration of lightweight or end-to-end trainable selection mechanisms. Model instability in extremely low-shot regimes (e.g., 1-shot) remains a challenge inherent to FSL, indicating the requirement for more robust regularization or structural constraints. The current study focuses primarily on classification. Extending the probe-then-adapt paradigm to structured prediction tasks, such as few-shot detection or semantic segmentation, would broaden its applicability.

Scalability deserves further study, and applying the proposed mechanism to larger medical foundation models may unlock stronger diagnostic capabilities. Developing meta-learning schemes that automatically infer critical hyperparameters (e.g., $K_s$ and $r$) from task characteristics remains an attractive direction for fully adaptive end-to-end systems.

### Clinical impact

HPF-Net’s task-adaptive capabilities suggest its promising clinical application. For novel data-scarce diagnostic tasks, the mechanism can quickly adapt internal representations from minimally labeled examples to deliver useful preliminary screening suggestions. The parameter-efficient design facilitates deployment in resource-constrained clinical settings, assisting to democratize AI-assisted diagnostics and alleviate the diagnostic workload at the point of care.

## Data Availability

All datasets used in this study are from the publicly available MedIMeta benchmark. No additional data were generated.

## Code availability

The source code will be publicly released upon acceptance.

## Abbreviations

Aml: Acute myeloid leukemia cell morphology dataset
AI: Artificial intelligence
AUROC: Area under the receiver operating characteristic curve
BitFit: Bias-term fine-tuning
Derm: Dermoscopy dataset
Dr_regular: Diabetic retinopathy grading dataset
EPT: Embedded prompt tuning
FSL: Few-shot learning
HPF-Net: Hierarchical probing and fusion network
LeakyReLU: Leaky Rectified Linear Unit
LoRA: Low-rank adaptation
MedIMeta: Medical imaging meta-dataset
Oct: Optical coherence tomography dataset
PEFT: Parameter-efficient fine-tuning
ViT: Vision transformer
VPT-S: Shallow visual prompt tuning
VPT-D: Deep visual prompt tuning ﻿
